# Supplementary figures and images for: Regulators of cell movement during development and regeneration in Drosophila
Source: Open Biol. 2019 May 1;9(5):180245. doi: 10.1098/rsob.180245 (PMC6544984; doi:10.1098/rsob.180245)

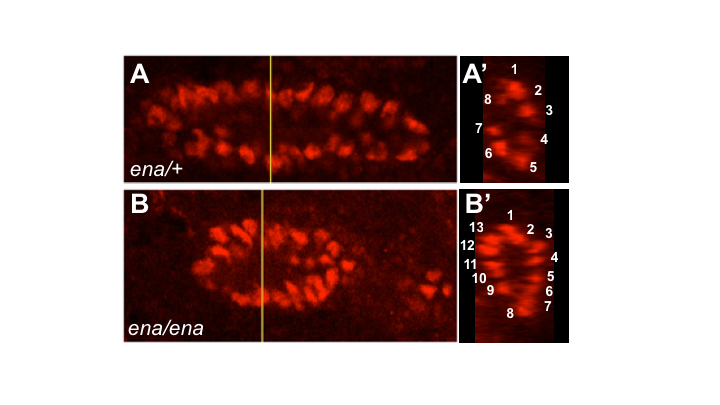

Supplement: Supplemental Figure S1 [file rsob180245supp1.tiff]
