# Supplementary material for: Regulators of cell movement during development and regeneration in Drosophila
Source: Open Biol. 2019 May 1;9(5):180245. doi: 10.1098/rsob.180245 (PMC6544984; doi:10.1098/rsob.180245)
Supplement: Supplemental Table S1. [file rsob180245supp2.pdf]

Table 1. RNAi lines used

CG	Gene name or Flybase ID	Flybase ID	Flybase symbol	Known or predicted molecular function	VDRC transformant ID	Salivary gland defects after RNAi with fkh-GAL4, this study	RNAi phenotypes from the literature				
							RNAi against core kinases, assayed for morphological defects in 6 cell lines. Defects seen in lines listed below (Liu et al., 2009, Ref15)	RNAi phenotypes in S2R+ and/or Kc cells (Kiger et al., 2003, Ref14)	RNAi phenotypes in S2R+ cells; genome-wide screen (Rohn et al., 2011. Ref16)	RNAi phenotypes in S2 cells (D'Ambrosio & Vale, 2010, Ref13)	Other screens
1227	…	FBgn0037491	CG1227	protein kinase	105610	No defect	S2R+ cells		…	…	
**1389**	**torso**	**FBgn0003733**	**tor**	**receptor tyrosine kinase**	**36280**	**shape/lumen defect**	S2R+ , BG2-c2 and BG3-c2 cells		…	…	
1768	diaphanous	FBgn0011202	dia	actin binding	103914	inconclusive		binucleate cells, F-actin accumulation and polarization, all in both cell types			cytokinesis failure (Ref53)
**1900**	**Rab40**	**FBgn0030391**	**Rab40**	**GTPase**	**110563**	**Migration defect**		Round, detached S2R+ cells	…	…	
2794	…	FBgn0031265	CG2794	hydrolase	13369	No defect	BG3-c1 and BG3-c2 cells		…	…	
**3086**	**MAP kinase activated protein-kinase-2**	**FBgn0013987**	**MAPk-Ak2**	**protein kinase**	**3171**	**Migration defect**		Round, detached cell S2R+ cells	…	…	
4027	Actin 5C	FBgn0000042	Act5C	actin/cytoskeletal	101438	No defect			smaller; decreased actin level; round or non-adherent; decreased actin level; multi-nucleate; loss of monolayer	toxic (reduced cell no.)	
**5179**	**cyclin dependent kinase 9**	**FBgn0019949**	**Cdk9**	**protein kinase**	**103561**	**Migration defect**	S2R+ and Kc cells	Round, detached cell shape in S2R+ and Kc cells	variable shape; decreased actin level; reduced cell no.; loss of monolayer	…	
5837	HEM-protein	FBgn0011771	Hem	Rac GTPase-binding	103380	No defect			Cell shape processes or spiky or stretchy; increased actin puncta; Decreased peripheral actin; loss of monolayer	stellate cells	
6148	Putative Achaete Scute Target	FBgn0016693	past1	calcium/GTP-binding	22253	No defect			…	…	endocytosis and membrane biology (Ref38-39)
7528	Ubiquitin activating enzyme 2	FBgn0029113	Uba2	Ub/SUMO-activating	110173	No defect			…	…	endocytosis (Ref52)
8465	Ankyrin repeat and LEM domain containing 2	FBgn0028343	Ankle2	protein phosphatase 2A binding	24107	No defect			…	nonspreading cells	
**8556**	**Rac2**	**FBgn0014011**	**Rac2**	**GTPase**	**28926**	**Migration defect**			…	stellate cells	
8711	Cullin 4	FBgn0033260	Cul4	Ub ligase binding	105668	inconclusive			shape variable; decreased actin; microtubule disorganized; decreased cell number; loss of monolayer		
8789	wallenda	FBgn0036896	wnd	MAP kinase kinase	103410	No defect			…	…	RNAi screen for cell invasion in wing discs (Ref55)
**8844**	**NADH dehydrogenase (ubiquinone) PDSW subunit**	**FBgn0021967**	**ND-PDSW**	**NADH dehydrogenase**	**106095**	**Migration defect**			shape variable; decreased peripheral actin; microtubule clumps; loss of monolayer	…	
9012	Clathrin heavy chain	FBgn0000319	Chc	clathrin light chain binding	23666	No defect			Cell shape processes or spiky or stretchy; Increased number of actin stress fibers; Increased peripheral actin; microtubules disorganized; loss of monolayer	…	
**9575**	**Rab35**	**FBgn0031090**	**Rab35**	**GTPase**	**101363**	**Migration defect**		binucleate S2R+ cells	…	…	
9765	transforming acidic coiled-coil protein	FBgn0026620	tacc	microtubule binding	101439	No defect			…	…	spindle function in early embryo (Ref57)
9881	Actin-related protein 2/3 complex, subunit 5	FBgn0031437	Arpc5	actin-binding	102012	No defect			…	stellate cells	
9901	Actin-related protein 2	FBgn0011742	Arp2	actin-like, cytoskeleton	1019999	No defect			…	stellate cells	
9985	skittles	FBgn0016984	sktl	Phosphatidylinositol 4-Phosphate-5 kinase	101624	No defect			Cell shape processes or spiky or stretchy; increased number of actin puncta or dots; decreased peripheral actin; loss of monolayer	…	
10540	capping protein alpha	FBgn0034577	cpa	actin filament binding	100773	No defect		F-actin accumulation and variable cell size/shape in both cell types	increased level of actin; multiple layers of cells	nonspreading cells	
10545	G protein β-subunit 13F	FBgn0001105	Gβ13F	Guanine nucleotide-binding	100011	No defect		F-actin accumulation, polarization, both cell types	…	…	
13623	…	FBgn0039205	CG13623	iron sulphur binding	110643	No defect			increased number of actin puncta or dots; asymmetric lamellae	…	
**15112**	**enabled**	**FBgn0000578**	**ena**	**actin binding**	**106484**	**Migration defect**		loss of actin filaments from the cell cortex, cell shape change	…	…	
31152	rumi	FBgn0086253	rumi	glycosyltransferase	14480	no defects					trafficking defects (Ref56)
32666	Death-associated protein kinase related	FBgn0052666	Drak	protein kinase	107623	No defect			…	…	Epithelial morphogenesis defects in the larvae (Ref54)
34361	Diacyl glycerol kinase	FBgn0085390	Dgk	diacylglycerol kinase	105753	No defect	S2R+ and Kc cells		…	…	
**42667**	**retinal degeneration A**	**FBgn0261549**	**rdgA**	**diacylglycerol kinase**	**28557**	**Migration defect**	BG3-c2 cells		…	…	
